# Energy metabolism during the perioperative period of gastric endoscopic submucosal dissection

**DOI:** 10.3164/jcbn.17-16

**Published:** 2017-08-18

**Authors:** Daisuke Chinda, Tadashi Shimoyama, Shiro Hayamizu, Kuniaki Miyazawa, Tetsu Arai, Miyuki Yanagimachi, Toshiaki Tsukamoto, Tatsuya Mikami, Shinsaku Fukuda

**Affiliations:** 1 Department of Gastroenterology and Hematology, Hirosaki University Graduate School of Medicine, 5 Zaifu-cho, Hirosaki, Aomori 036-8562, Japan; 2 Department of Endocrinology and Metabolism, Hirosaki University Graduate School of Medicine, 5 Zaifu-cho, Hirosaki, Aomori 036-8562, Japan; 3 Department of Rehabilitation, Hirosaki University Hospital, 5 Zaifu-cho, Hirosaki, Aomori 036-8562, Japan; 4 Division of Endoscopy, Hirosaki University Hospital, 5 Zaifu-cho, Hirosaki, Aomori 036-8562, Japan

**Keywords:** endoscopic submucosal dissection, energy metabolism, early gastric cancer, perioperative period, indirect calorimeter

## Abstract

The aim of this study was to investigate the change in the energy metabolism and invasiveness in the perioperative period of endoscopic submucosal dissection for early gastric cancer. Fifty-two consecutive patients were enrolled into the study between July 2013 and May 2014 and examined resting energy expenditure using an indirect calorimeter, body weight and basal energy expenditure using the Harris–Benedict equation before and after endoscopic submucosal dissection. Resting energy expenditure/body weight and resting energy expenditure/basal energy expenditure were 20.2 ± 3.0 kcal/kg/day and 0.96 ± 0.11 on the day of endoscopic submucosal dissection, whereas one day after the endoscopic submucosal dissection they were 21.7 ± 3.2 kcal/kg/day and 1.03 ± 0.14, showing significant increases (*p*<0.001, respectively). The stress factor on the postoperative day 1 was computed as 1.07. This increase was low in comparison to that experienced for surgery, suggesting that the degree of perioperative invasiveness in patients receiving endoscopic submucosal dissection is lower in comparison to that during surgery (The study of the resting energy metabolism and stress factor using an indirect calorimeter in the perioperative period of endoscopic operation: UMIN000027135).

## Introduction

Endoscopic submucosal dissection (ESD) is a highly safe and useful procedure in the treatment of early gastric cancer.^(1–5)^ Compared to conventional endoscopic mucosal resection, ESD has high en bloc resection rates and en bloc curative resection rates; ESD also allows for accurate histopathological search.^(6)^ ESD has been adopted even in cases where surgery was previously common for early gastric cancer lesions, resulting in curative resection in many cases.^(7)^ In comparison with surgery, the biggest advantage of ESD is that it is less invasive for the patient; as a result, it is a treatment method of choice for the elderly in whom surgery is deemed difficult.^(8,9)^ Along with the increase of elderly people, the number of patients who undergo ESD for gastric cancer is increasing. However, since no studies have been conducted on physical stress and the change in the energy metabolism in patients during the ESD perioperative period, the range and degree of stress remains unclear as it relates to ESD.

Reports show that the increase in salivary amylase activity in patients undergoing ESD for early gastric cancer may be a sensitivity marker for intraoperative pain stress.^(10)^ However, this change is an ultra-acute reaction by the endocrine system; thus, it cannot be used to evaluate patient stress entirely during the preoperative period of ESD.

On the other hand, it is known that in the perioperative period of surgery, metabolism varies greatly due to invasion, and energy requirements increase due to the increase in stress.^(11–17)^ Moreover, one of the quantitative markers of invasiveness during the perioperative period is the change in stress factor. Previous studies have reported on the increase in calorie consumption and stress factor in surgery for gastric cancer, but no such study has been reported pertaining to ESD.

In the present study, with respect to invasiveness in the ESD perioperative period for early gastric cancer, resting energy expenditure (REE) was measured before and after ESD using an indirect calorimeter, and the change in amount of energy required based on body weight (BW, kg) was investigated. The stress factor was computed using the REE and basal energy expenditure (BEE), and the variation between preoperative and postoperative ESD states was studied.

## Materials

Patients who underwent ESD in the hospital affiliated with Hirosaki University School of Medicine from July 2013 to May 2014 were prospectively studied. ESD was performed using a conventional single channel endoscope (GIF-Q260J or GIF-H260; Olympus, Tokyo, Japan) with hood. The ESD procedure was mainly performed using a water jet short needle knife (Flush Knife BT-S; DK2620J, Fujinon, Tokyo, Japan) and Hook Knife (KD-620LR, Olympus). We also used a high frequency generator with an automatically controlled system, ICC200 and VIO300D thereafter (both supplied by ERBE, Tübingen, Germany). In all patients, we initially used pethidine hydrochloride (25–100 mg/body), together with midazolam or diazepam. All ESD procedures were peformed by Board Certified Endoscopists of The Japan Gastroenterological Endoscopy Society.

**Fig. 1:**
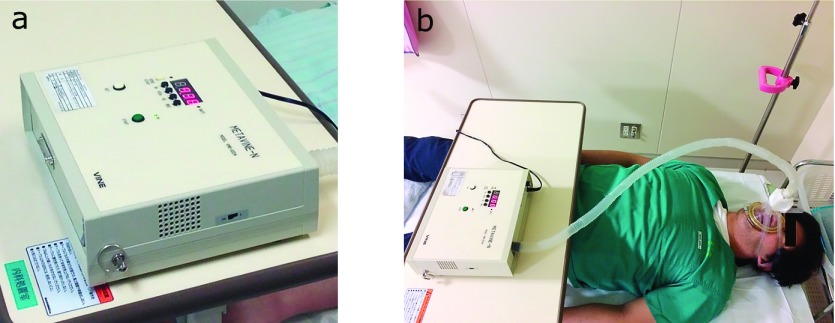
The actual image of METACINE-N. (a) METAVINE-N VMB-002N. (b) Measurement by METAVINE-N.

The REE was measured using indirect calorimeter METAVINE-N VMB-002N (VINE, Tokyo, Japan) (Fig. 1). METAVINE computes the REE using the oxygen concentration and respiration rate in the breath; it does not use the carbon dioxide concentration.^(18)^ Since respiratory quotient remains approximately constant at the time of rest, and the inter-individual differences are also considered to be small, REE can be measured assuming a constant respiratory quotient under bed-rest. Each patient fasted for more than 12 h the previous night, and on the day of ESD, REE was measured after 30 min of bed-rest early in the morning. Subsequently, ESD was conducted; upon completion and without any food intake, REE was measured once again on the next day early in the morning. REE was measured three times, and was computed as the mean when the variations were less than 100 kcal. When the variations were >100 kcal, a fourth measurement was taken and REE was computed as the mean from three measurements, excluding the one that was further from the mean of the two median values. Some patients were nervous at first and breathed in deep or shallow bringing unstable results. In other cases, results were not stable because of insufficient setting of the mask. Therefore, we increased the number of measurements to obtain stable results as we could. On the other hand, BW was measured prior to ESD and within three days after ESD.

From these measurements, the change in REE per kg of body weight (REE/BW) between preoperative and postoperative ESD states was investigated. In addition, BEE was estimated using the Harris–Benedict equation based on the height and BW of each patient.

Moreover, according to Long’s method,^(19)^ the total energy expenditure can be determined by multiplying BEE with activity and stress factors. In theory, this value is the same as REE measured in a state of rest, and stress factors are markers of a hyper-metabolic status.^(20–24)^ Accordingly, the change in stress factors was investigated by computing REE/BEE on the day of ESD and on the postoperative day 1.

This study was approved by the Hirosaki University ethics committee. Prior to the admission or on the day before ESD procedure, the details of the investigation procedure and the research objective were explained to the participants and written consent was obtained from all participants who were willing to collaborate.

In the analysis, continuous variables were expressed as mean ± SD. Paired *t* test was used for comparison of REE, BW, REE/BW, and stress factors between preoperative and postoperative ESD states, and a *p* value less than 0.05 was considered statistically significant using SPSS ver. 24. OJ (SPSS Inc., Chicago, IL).

## Results

### Patients’ characteristics

**Fig. 2:**
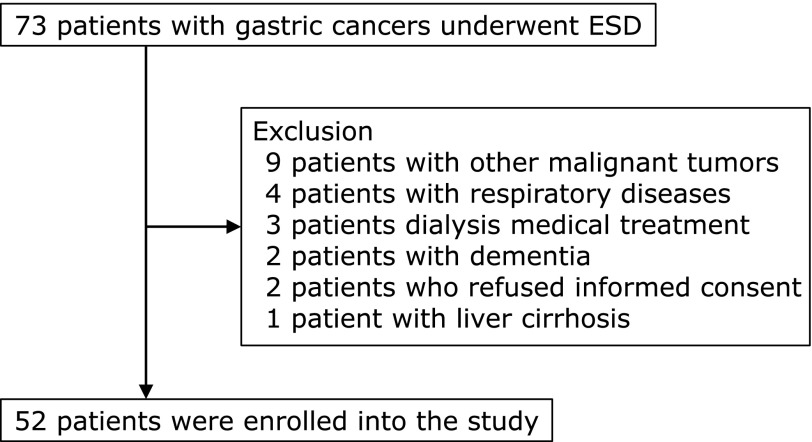
Flowchart of this study.

All patients who underwent ESD from July 2013 to May 2014 were seventy-three. Fifty-two patients in all were examined, excluding patients with cirrhosis of the liver, respiratory disease, or who were undergoing dialysis medical treatment or other malignant tumor medical treatment, since such disorders affect the measurement of indirect energy requirements (Fig. 2).^(25–27)^ Sample size was calculated using significance level (α) of 0.05 and power (1 – β) of 0.80. In results, a sample of 52 was statistical power 0.991 (stress factor) and 0.982 (REE/BW), and it was considered good.

The breakdown of patients is shown in Table 1. Therefore, the operation period included not only the operation period of ESD but also the introduction period of sedation and observation time of the lesion. It should be noted that the resection area was computed by approximating it as an ellipse with the length and breadth of the resection specimen, and for 16 patients with multiple lesions, the total resection area of all the lesions was computed. With respect to complications after procedure, perforation occurred in one case, postoperative bleeding in two cases, and fever >38°C in two cases were observed; all patients received successful medical treatment with no patient needing additional surgical treatment for complications.

### REE and BW

The changes in REE and BW in the perioperative period of ESD are shown in Table 2. On the day of ESD, the REE was 1,170.3 ± 209.0 kcal, whereas the next day, it was elevated to 1,238.4 ± 235.5 kcal (*p*<0.001). In contrast, with respect to BW, there was a significant reduction from 59.0 ± 13.0 kg to 58.0 ± 12.3 kg between the ESD preoperative and postoperative states, respectively.

### REE/BW

**Fig. 3:**
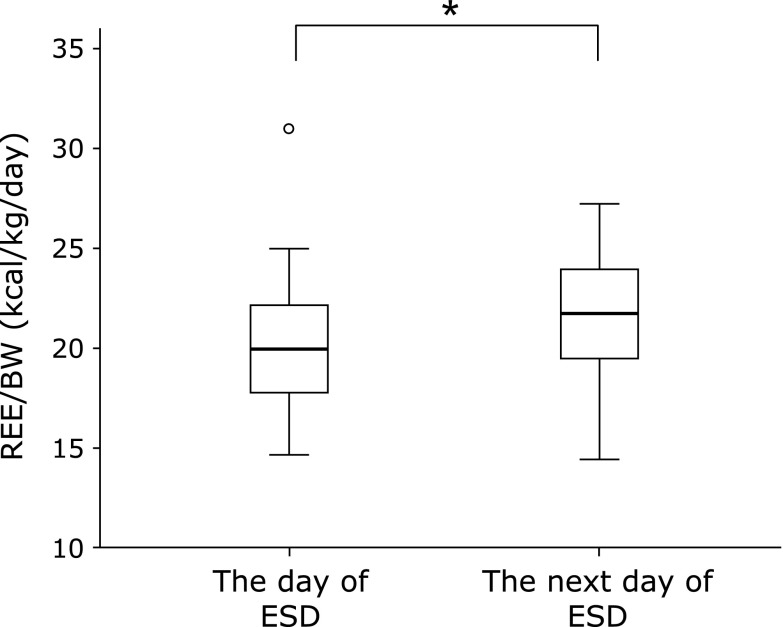
The change in resting energy expenditure/body weight (REE/BW) during the perioperative period of endoscopic submucosal dissection (ESD). Data are shown as mean ± SD. *p<0.001: compared with the value of the day of ESD.

The REE/BW was elevated in 41 (78.8%) out of 52 patients. REE/BW was 20.2 ± 3.0 kcal/kg/day on the day of ESD, whereas one day after the ESD it was 21.7 ± 3.2 kcal/kg/day, showing a significant increase (*p*<0.001, Fig. 3).

### REE/BEE

**Fig. 4:**
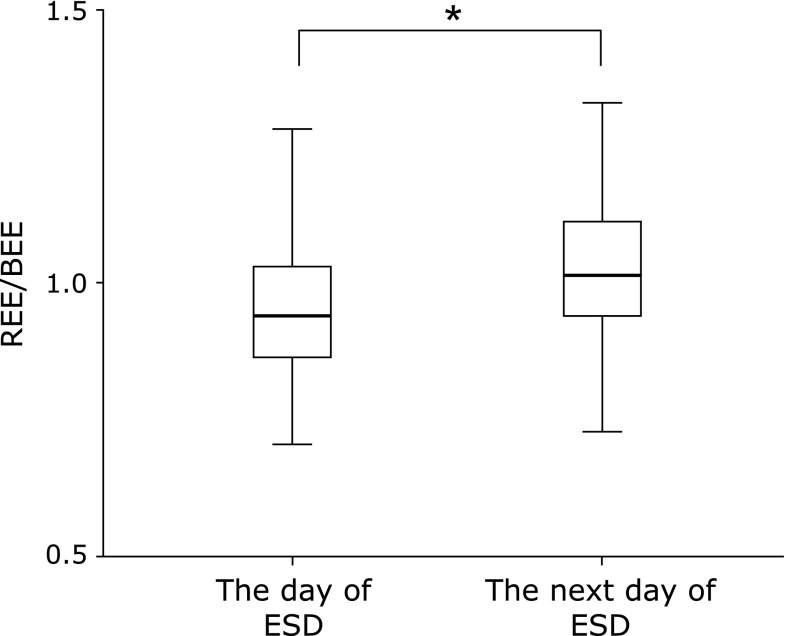
The change in energy expenditure/basal energy expenditure (REE/BEE) during the perioperative period of endoscopic submucosal dissection (ESD). Data are shown as mean ± SD. *p<0.001: compared with the value of the day of ESD.

REE/BEE was elevated in 39 (75%) out of 52 patients. On the day of ESD, REE/BEE was 0.96 ± 0.11, whereas on the next day it was 1.03 ± 0.14 showing a significant increase (*p*<0.001, Fig. 4).

## Discussion

This is the first study to assess REE changes in patients during the ESD perioperative period. The energy requirements increase in association with the degree of invasiveness to the living body under pathological stress (due to surgery, severe burns, trauma, sepsis, malignancy, etc.).^(13–17,28,29)^

In indirect calorimetry, oxygen consumption and carbon dioxide production in the oxidation process of the energy substrate are measured to indirectly compute the energy expenditure.^(14,20,21)^ REE is compared with BEE computed using the Harris–Benedict equation to reflect the energy metabolism state of each individual patient.^(19)^ In the present study, during the perioperative period of ESD, REE/BW was 7.3% higher on the postoperative day 1 in comparison to the day of ESD. It is likely that this is related to an increase in REE and decrease in BW.

In many of the previous studies on increase in energy expenditure during the surgical perioperative period, comparisons of BEE based on the Harris–Benedict equation have been reported,^(26)^ but very few REE measurements have been reported. It is likely that, in comparison to BEE, REE measurements are more laborious and complex, and very few facilities are equipped with calorimeters. With respect to REE in preoperative and postoperative gastric cancer surgery, Fredrix *et al.*^(11)^ have reported that in gastric and colorectal cancer surgery, REE on postoperative days 7 and 8 was 106.9% of the corresponding preoperative value. Moreover, in Japan, in a study on energy expenditure changes during the perioperative period of gastric cancer surgery, Yoneyama *et al.*^(30)^ reported that, as opposed to the preoperative REE/BW value of 21.75 ± 1.46, the postoperative day 1 value increased by 26.5% to 26.06 ± 5.30. They surmised that this increase in energy expenditure could be attributed to surgery invasion. In the present study, in the ESD perioperative period, REE/BW was elevated in many of the patients. However, in previous studies, REE was only compared in the curative period one week after surgery; however, considering that in the present study, REE was measured on the postoperative day 1 when the degree of invasion is likely to be at its highest, an increase of 9.2% is considerably low.

Both REE/BW and REE/BEE have been used to evaluate energy metabolism. REE/BW and REE/BEE are similar, but not the same. Because formula to calculate BEE is different between male and female. Indeed, in this study, REE/BW was elevated in 41 out of 52 patients (78.8%), whereas REE/BEE was elevated in 39 patients (75.0%). Previous reports have evaluated the invasiveness of surgical operation by one of REE/BW and REE/BEE. Therefore, we had to calculate both REE/BW and REE/BEE to compare our results with those of previous studies for surgical operation.

With respect to stress factor, Long *et al.*^(19)^ have reported it to be 1.1 for low invasion, 1.2 for medium invasion, and 1.8 for high invasion, and these have been used as guidelines for determining energy requirements and administration in long-term perioperative surgery periods. However, with recent progress in surgical procedures and perioperative management, it has become feasible to undertake procedures less invasive to the patients, and attempts are being made to compute stress factors for more specific procedures. In Japan, in a study involving gastrectomy, Inoue *et al.*^(31)^ have reported a stress factor of 1.4 on the postoperative day 3 of a medium-degree invasion such as subtotal gastrectomy, and 1.6 on the postoperative day 3 of a high-degree invasion such as total gastrectomy. In the present study with respect to endoscopic treatment, REE was measured on the postoperative day 1 when the physical and mental burden on the patient was likely to have been the greatest. The results suggest a significant elevation of REE/BEE on ESD postoperative day 1. Since the preoperative and postoperative activity factors are the same, assuming the REE/BEE on the day of ESD to be 1, the REE/BEE measured on the postoperative day 1 can likely be considered the stress factor. Based on this assumption, the stress factor on ESD postoperative day 1 was computed as 1.07, and remained low in comparison to that of the aforementioned cases of distal gastrectomy or total gastrectomy. Recently developed surgical techniques such as laparoscopic surgery, function-preserving surgery, and laparoscopic and endoscopic cooperative surgery are performed for the treatment of gastric cancer. These treatments are also less-invasive in comparison with open surgery. Further studies comparing the invasiveness between ESD and the less-invasive surgeries would be desirable.

Among the 52 patients in the study, there were some complications: perforation was observed in one case, postoperative bleeding in two cases (postoperative days 1 and 9), and fever of >38°C in two cases (postoperative days 1 and 4). However, since the number of cases was so low, they were not considered to be contributing complication factors for an increase in stress factor. We believe that detailed studies will be required to accumulate more data in a variety of further cases.

This study has some limitations. One is that this study was carried out in a single institute. Indirect calorimeter is a very expensive equipment and our hospital is only one institution which has indirect calorimeter in our area. Another limitation is that this is a single arm study without healthy control or surgical patients. It was not possible to perform the same measurement on healthy subjects who underwent non-therapeutic endoscopy. Since we did not assess surgical patients, we could only compare the change of energy metabolism of ESD to those of open surgery in previous observations. Recently laparoscopic surgery and minimally invasive surgery are widely carrying out. Further multi-center studies are required to compare the change energy metabolism between ESD and minimally invasive surgery.

In the present study, the stress factor on ESD postoperative day 1 was 1.07 and energy expenditure per kg of body weight and stress factor increased during the ESD perioperative period for early gastric cancer. However, this increase was low in comparison to that experienced for surgery, suggesting that the degree of invasiveness in patients was low during the ESD perioperative period in comparison to that during surgery.

## Figures and Tables

**Table 1:** Basic characteristics

Parameter	Value
Total patients	52
Age (years old)^a^	70.2 ± 8.1 (52–83)
Sex (Male:Female)	39:13
Multiple lesion’s cases^b^	14 (26.9%) (×2: 10, ×3: 3, ×5: 1)
Operation time (min)^a^	130.6 ± 66.5 (31–331)
Total resection area (cm^2^)^a^	15.2 ± 12.2 (3.1–68.7)
The maximum length of main 52 lesions (cm)^a^	2.1 ± 0.8 (0.4–3.8)
The existence of ulcerative change^b^ (Total 72 lesions)	13 (18.1%)
Histopathological diagnosis of the total 72 lesions^b^	
Indication lesion	42 (58.3%)
Extended indication lesion	22 (30.6%)
Non-indication lesion	8 (11.1%)
Complications^b^	
Perforation	1 (1.9%)
Bleeding	2 (3.8%)
Fever (>38.0°C)	2 (3.8%)

^a^Data are presented as mean ± SD (range). ^b^Data are presented as number of positive case (percentage).

**Table 2:** The change of resting energy expenditure (REE) and body weight (BW) during the perioperative period of endoscopic submucosal dissection (ESD)

	The day of ESD	The next day of ESD	
REE (kcal)	1,170.3 ± 209.0	1,238.4 ± 235.5	*p*<0.001

	ESD preoperative state	ESD postoperative state	
BW (kg)	60.0 ± 13.0	58.0 ± 12.3	*p*<0.001

Data are presented as mean ± SD.
